# A formative journal for a formative career: a personal recollection of how *JCPA* has inspired and guided my research life

**DOI:** 10.1007/s00359-023-01683-1

**Published:** 2023-12-11

**Authors:** Eric J. Warrant

**Affiliations:** Lund Vision Group, Department of Biology, University of Lund, Sölvegatan 35, 22362 Lund, Sweden

**Keywords:** Entomology, Physics, Optics, Superposition compound eye, Insect

## Abstract

A fateful decision as a 15-year-old high school student, and good advice from a distinguished professor of zoology, were the catalysts that not only decided my entire career but also led me to the *Journal of Comparative Physiology A*, and to the myriad biological wonders that were held within its covers. In my celebration of *JCPA*, I look back on the formative years of my career in Australia, and the crucial role that the journal played in shaping my emerging research interests, and ultimately my entire life.

I remember the moment very clearly. It was the middle of 1984, and I was nearing the end of my undergraduate science degree at the University of New South Wales in Sydney. I was standing in the corridor outside the office of the Professor of Zoology, renowned crustacean neurobiologist David Sandeman, with a burning question that had plagued me for at least a year. His answer to that question would change my life forever and set me on a course that I still follow today. But before I get to that question—and why I even needed to ask it—some background is required.

I was fortunate to grow up on the edge of a coastal rainforest just north of Sydney. It was an idyllic childhood, and the rainforest was a magical world of secret tracks through ferns to makeshift hideouts (which Australian kids call “cubbies”), where thick vines hanging from the boughs of massive eucalypts swung us precariously out over deep ravines and creeks (our mothers never knew!), of clouds of screeching rosellas racing through the dense canopy above and of the incessant soft pealing of bell birds, whose calls mimic the ringing of tiny bells. But above all it was a world of insects, and I became increasingly fascinated by them. Massive stick insects, ogre-headed leaf insects with near-perfect camouflage, bottle-green praying mantises longer than a spoon, terrifying robber flies (still the biggest I have ever seen) that had a habit of menacingly circumnavigating your head, gorgeous jewel-like Christmas beetles and a myriad of spectacular butterflies. It soon became obvious to me that insects would become my life. When I grew up, I was going to be an entomologist.

Unfortunately, unbeknown to me at the time, another childhood passion would nearly derail that plan entirely. I was incredibly interested in art and art history (I still am), and I expanded this subject in my later high school years to the maximum possible. Stupidly, as it turned out, to the detriment of physics, which I simply skipped to fit in all that art. So imagine my surprise at the end of my last high school year, when I turned up to the open day of the University of New South Wales (the university in Sydney I had deemed best for studying entomology), to be told “Sorry, but you needed to have studied physics to begin a science degree here.” Noticing the utter horror that clearly spread across my face upon hearing this bombshell, the lady then kindly added “Ah but don’t worry, we have a solution for people (I heard “idiots”) like you. We run a three-month intensive course covering all of high school physics in the summer holidays before term starts. I suggest you take it.” I did.

Unfortunately, this only created the next dilemma. The quality of this course was so high, and the lecturers so excellent (many were world famous), that I entirely fell in love with physics. My urge for entomology suddenly had a competitor, and this unexpected new scenario caused a fair bit of pain. But then I had a crazy idea. I decided to create my own program of studies leading to a double major in physics and entomology, a seemingly absurd combination, and submitted it to the University senate in hope of their approval. Incredibly, to my immense surprise, they *did* approve! I was probably the first and the last whoever pursued this bizarre double major, but I have never regretted it for a second. To the contrary, it has served me incredibly well.

But back to that question. After nervously knocking on David Sandeman’s door, a friendly voice ushered me in. Sandeman ran a higher-level undergraduate course in invertebrate neurobiology that I had taken the term before, a subject that under his guidance had impressed me deeply. My question to him was short and simple: where could someone like me, with majors in so disparate topics as entomology and physics, go to do a PhD? To my enormous surprise, his answer was both swift and enthusiastic—the Australian National University in Canberra, and the lab of George Adrian Horridge. Horridge ran arguably the most successful research environment for studying the vision of arthropods in the world (not that I knew that at the time), and Sandeman had until a couple of years earlier spent years in this department. Here, he assured me, was the place for me. Horridge’s group, he told me, was full of engineers, mathematicians and physicists all trying to understand the neural and optical mechanisms that allow insects (in particular) to see, and the intellectual environment was world class. I am eternally indebted to David Sandeman for this (literally) life-changing advice. Six months later, with all my worldly possessions stuffed into my old car, I arrived in Canberra.

After 5 years in the smoggy car-mired bustle of downtown Sydney, Canberra seemed like heaven. Walter Burley Griffin and Marion Mahony Griffin, the American husband-and-wife team of landscape architects that won an international competition in 1912 with a revolutionary city design for Australia’s new post-Federation capital, had made sure that the Australian landscape and its animal-filled forests penetrated all parts of the city. The result is simply beautiful, and the campus of the Australian National University—especially when I arrived in early 1985—is a lush parkland of lawns, gardens, creeks and groves of majestic eucalypts. And lots of insects. It was love at first sight. Soon after arriving, I was placed in the care of Dr. Peter McIntyre, a mathematician who had just returned from a couple of years at the Max Planck Institute for Biological Cybernetics in Tübingen, where he had worked with Kuno Kirschfeld on the optics of fly eyes. Peter was now engaged in trying to understand the optics of superposition compound eyes, the type of compound eye, due to its enhanced ability to capture light, that is typical of nocturnal insects such as moths and beetles. Peter’s insects of choice for this work were several species of introduced South African dung beetles from the genus *Onitis*. These had been introduced over the previous two decades to reverse Australia’s enormous bovine dung problem, where millions of hectares of good pastures were rendered unproductive for months and flies bred in such explosive numbers that in some parts of the country outdoor restaurant dining was banned. Our native species had never evolved for these colossal wet dumps (cows not being native animals) and the introduction of beetles evolved over millennia to devour and eliminate such veritable piles of dung has since become one of the great success stories of Australian biological control.

This was all good news for me, as I quickly became engaged in Peter’s work as well. These different species of Onitine dung beetles could be found in good numbers all over southeast New South Wales (around Canberra), and each was adapted to a different light intensity niche, some day-active, others nocturnal and still others active at specific time (and thus light intensity) windows during dusk and dawn. And they all had superposition eyes. I soon embarked on trying to understand how superposition eyes worked, how their optics should be adapted for vision at different light levels and whether nocturnal superposition eyes truly had sacrificed the ability to resolve spatial detail to maximise light capture (as was believed at the time). I planned to attack these questions via theoretically modelling the optics of the eyes in different species and by experimentally measuring image quality electrophysiologically from photoreceptors in the retina.

But first I needed to learn about insect eyes and insect vision and immerse myself in everything we knew about superposition eyes. I spent hours in our departmental and university libraries. Adrian Horridge had the good foresight to regularly collect all reprints in the field of insect vision as they emerged, cataloguing them, categorising them and having them bound into wide and heavy volumes embossed in gold on the spine to aid searching. It was an amazing resource. What became immediately apparent during my deep diving into the literature was the prominent, no dominant, role that the *Journal of Comparative Physiology* (and later *Journal of Comparative Physiology A*, or *JCPA*) had (and was) playing in the development of our field. It seemed that everyone that was anyone in the field of insect vision was publishing their work in the journal. And for me, trying to understand the optics and designs of compound eyes, it was all there. Whether it was the optical properties of the rhabdoms (Allan Snyder, Simon Laughlin, Hans van Hateren and Doekele Stavenga), the optics of the entire eye (Mike Land, Joe Howard and Dan-Eric Nilsson), the properties of pupillary screening pigments (Stavenga) or the ability of a compound eye to transmit information (Snyder, Laughlin, Stavenga and van Hateren), *JCPA* had it all. Later in my PhD I discovered many other riches in *JCPA*, including the remarkable (and for me life-changing) concept of sensory matched filters (Rüdiger Wehner) and the amazing ways that arthropods orient and navigate (Wehner, Jochen Zeil, Tom Labhart, Tom Collett and Mandyam Srinivasan). No other journal has had the impact on me that *JCPA* has had. There is no question—it was a formative journal for a formative career. It is hardly a surprise that all five papers that resulted from my PhD thesis were published there (Warrant and McIntyre 1990a, b, 1991, 1996; Warrant et al. 1990).

It did not take long after getting to Canberra that I realised that many of my new “*JCPA* heroes” had been there before me—in fact only until a month or two before I arrived! How on Earth could this be true? Dan-Eric Nilsson and Mike Land, both of whom had a profound influence on me, had just finished a couple of years in Canberra before returning to Lund and Sussex, respectively. Others had literally been in Canberra for decades—like Simon Laughlin and Roger Hardie. They had just gone back to Cambridge. I did think when I arrived in Canberra that it felt a little deserted. When I realised who had been there, that feeling only deepened! Some were still around—like the wonderfully eccentric Allan Snyder—and soon “Srini” Srinivasan would arrive from Switzerland to continue his outstanding research on honeybee flight control and navigation. Adrian Horridge, and Canberra, were magnets for everyone in the field, and soon his department began to fill with fresh arrivals (Fig. 1). The tearoom discussions began to buzz with the influx of new people and ideas, and soon lots of new PhD students (and new friends) arrived. I set about building an electrophysiology lab from equipment I scrounged from various others in the group, and soon Adrian challenged me and another new student—Andrew James—to a competition to see who would be the first to obtain intracellular recordings of light responses from the photoreceptor of a locust. Luckily, this seemingly impossible task was made easier by Adrian’s delightful and generous electrophysiology technician Ljerka Marcelja who introduced Andrew and I into the mysteries of these difficult methods. Needless to say, Andrew beat me to the first recording, but it was not long before I had my first recording as well. In between bouts in the lab, I had my head in the literature, and my fascination for insect vision grew by the day. This period of my life was one of my happiest, without a care in the world and left only to do my research. And *JCPA* was a huge part of all of it.

**Fig. 1:**
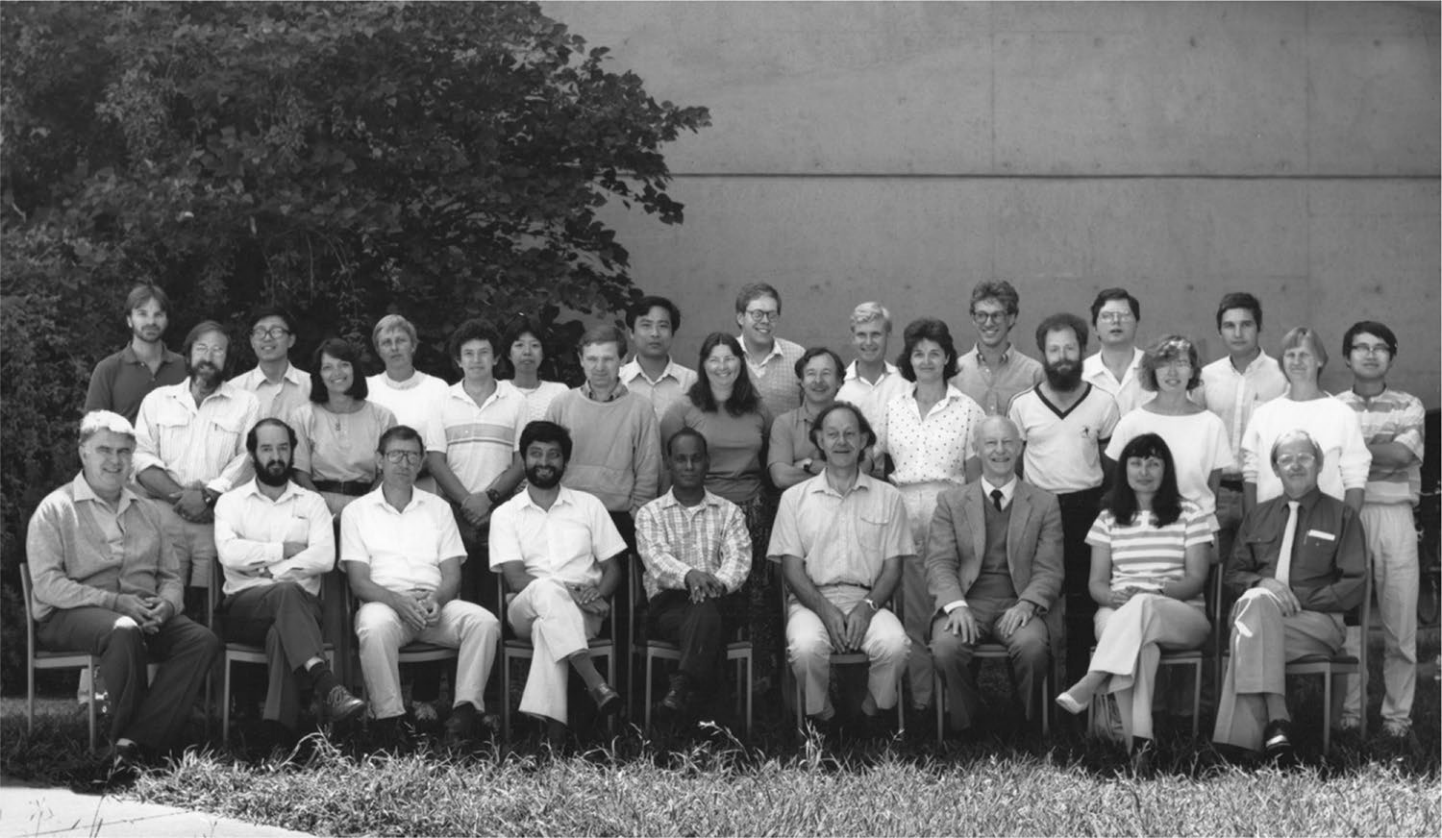
Various members and friends of the Department of Neurobiology, Research School of Biological Sciences, Australian National University (at the opening of the ANU Centre for Visual Sciences in 1987), showing some of the people who mentored and inspired me: my supervisors Professor George Adrian Horridge (seated, fourth from right) and Professor M. V. “Srini” Srinivasan (seated, fourth from left), Professor Robert Pinter on sabbatical from the University of Seattle (standing, second from left), with whom I shared an office and a close friendship, and Ljerka Marcelja who patiently and graciously taught me electrophysiology (standing, fourth from left). I can be seen standing at the back (ninth from the right). Other notable people in this picture include Professors Geoff Henry (seated, far left) and Bill Levick (seated third from right), well-known figures in mammalian vision from the John Curtin School of Medical Research (Levick was particularly famous for the Barlow-Levick model of visual motion detection), Dr. Peter Coombe (standing, sixth from the right), one of the pioneers of recording from monopolar cells in the fruit fly lamina, fellow PhD student (now ANU Professor) Ted Maddess (who became famous for his methods for treating glaucoma—standing, fifth from the right) and Dr. Wolfgang Kirchner (standing, third from the right), who later returned to a professorship in Germany. Mike Savage (standing, far left) ran the electronic workshop and kept me supplied with all manner of devices for my new lab, and Lillian Chan (standing, seventh from the left), was our intrepid typist who somehow miraculously managed to transform our messy stacks of hand-written pages into submittable manuscripts. Those were the days! Photograph copyright: Adrian Horridge

Soon I found out about Mike Land’s incredible descriptions of the strange and wonderful optical mechanisms of eyes in a huge variety of invertebrates, both aquatic and terrestrial. Among the first of his papers I read in *JCPA* were those on the optical geometries of compound eyes from different species of marine crustaceans living at different depths in the ocean, notably on the apposition eyes of amphipods (Land 1981, 1989) and the superposition eyes of euphausiids (Land et al. 1979). What a feast of optics! Such extraordinary variation in optical design matched to the predicable light environment of the open ocean. Near the surface daylight is fairly bright in all directions, leading to “all-round” eye designs where decent spatial vision is uniformly spread within the visual field of the eye. But deeper down in the ocean, where the ever-diminishing daylight comes increasingly from above, and light arriving from other directions is orders of magnitude dimmer (particularly from below), compound eyes optically deform to concentrate their light capture and spatial resolving power to a narrow dorsal visual field, with those parts of the eyes pointing in other directions largely rudimentary. Indeed, in many species the eyes are physically divided, with a huge dorsal apposition or superposition eye pointing upwards, and a tiny ventral compound eye being left to capture light from other directions. One extreme dorsal superposition eye is found in the deep-sea euphausiid species *Stylocheiron maximum*, whose upward visual field is so narrow that the most appropriate retinal imaging plane becomes flat (Fig. 2A). Amazingly, the same flat retina can be found in the dorsally pointing camera eyes of fish from the same environment, an adaptation that has convergently evolved to match vision to this extreme habitat (Fig. 2B).

**Fig. 2:**
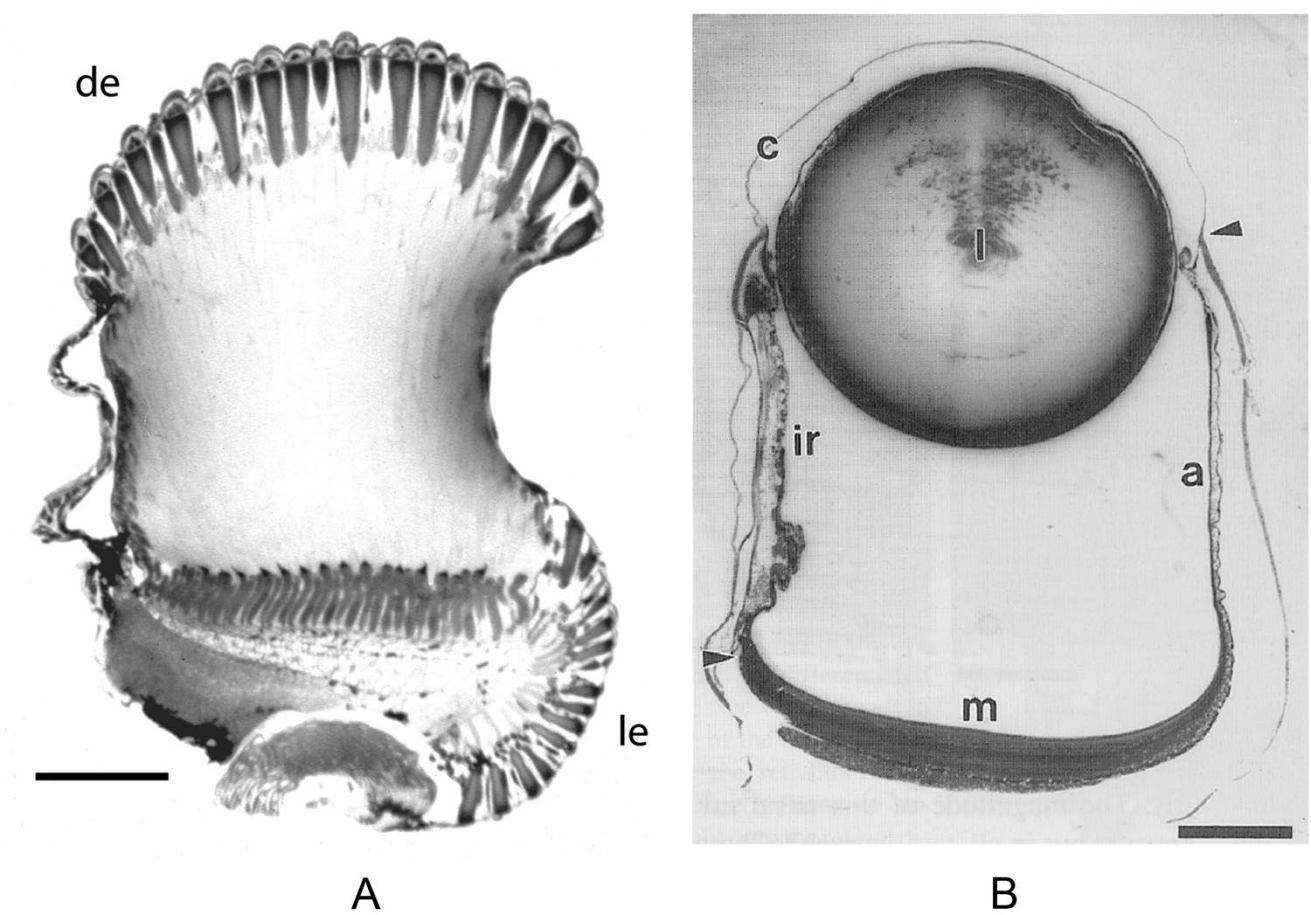
Convergent evolution of dorsally directed tubular eyes in the mesopelagic zone of the deep sea. **A** The divided superposition eye of the euphausiid shrimp *Stylocheiron maximum.* The huge dorsal lobe (de) has a narrow dorsal field of view for viewing the down-welling daylight, while the small lateral lobe (le) has a broader lateral field of view which could be useful for viewing bioluminescent flashes. Photograph courtesy of Dan-Eric Nilsson. Scale bar 200 μm. **B** Transverse section through a tubular eye of the hatchet fish *Opisthoproctus soleatus*, showing the main retina (m), the accessory retina (a), the spherical lens (l), and the iris (ir). The cornea (c) extends between the arrowheads. Scale bar: 0.5 mm. From Collin et al. (1997). Copyright © 2010 Karger Publishers, Basel, Switzerland

These early papers by Mike Land in *JCPA* made me realise that even though compound eyes have a basic design—a ball-like structure built from hundreds or thousands of individual ommatidia,^1^ each receiving and analysing light from a different direction—this basic design can be warped and moulded by evolution to optimise vision for different lifestyles and habitats. In other words, the designs of compound eyes evolve to become matched to the ecologies of their owners. This realisation that vision is ecological (and not only for compound eyes) was a watershed moment in my young career and changed the entire direction of my research for ever. Instead of being obsessed by their optics per se, these papers by Mike Land led to an obsession for what eyes are actually used for, and how their optics and visual processes are matched to these many uses—a research field known as “visual ecology”. In my case, this developed into a life-long interest for how eyes and visual systems have become optimised for vision in very dim light, notably in nocturnal and deep-sea habitats (Warrant 2004), a topic I have worked on for my entire career!

The myriad ways that the senses have evolved to become matched to the ecologies of animals is a truly beautiful and awe-inspiring topic within sensory biology. The astonishing breadth of amazing adaptations that have evolved to enhance the detection and capture of prey, to discern and outwit a predator or to woo a member of the opposite sex are frequently highlighted in natural history documentaries, including the magnificent productions of David Attenborough. It was such a pleasure to team up with Tom Cronin, Sönke Johnsen and Justin Marshall to write the first book on the topic of visual ecology since the 1970s (Cronin et al. 2014), and to edit a volume on the more general topic of sensory ecology with Gerhard von der Emde in 2016 (von der Emde and Warrant 2016). And *JCPA* was the beginning of it all!

At the same time Mike Land was revealing the optical splendours of marine crustaceans, Allan Snyder was creating the first physical description of how the imaging properties of the ommatidia, and their packing density in the eye, impact the amount of visual information that can be extracted from the world at different ambient light levels and locomotion speeds. Allan was a deeply fascinating and likeable individual, a brilliant and seriously eccentric professor from the Applied Mathematics department who was rumoured to sunbake naked in the departmental garden! A mixture of salesman, clown and genius, his nasal New York accent, his high-pitched laugh and his signature train-drivers cap were frequent and welcome fixtures in our tearoom. But despite all his eccentricities, I have seldom read mathematical accounts as clear as Allan’s on any topic. His physics and mathematics packed treatise on compound eye design appeared in *JCPA* in 1977 (Snyder 1977). To say I was blown away by this work would be an understatement. Here was the mathematical basis of visual ecology in compound eyes. For instance, how should a compound eye be built to work best in dim light, or in bright light if one flies fast? And how should ommatidial size and packing density vary throughout an eye to maximise information uptake during forward flight, or to intercept a target in the frontal visual field? As a physicist, this paper—together with a later paper in *JCPA* formalising the information capacity of compound eyes (Snyder et al. 1979)—had a profound impact on me, and deeply influenced my own theoretical studies on how compound eyes and visual processing should be organised to optimise visual performance in dim light (Warrant 1999).

But *JCPA* was still revealing other intellectual pleasures of a purely optical nature. On my own topic of superposition eyes, one in particular stands out, once again by Mike Land (Land 1984). During his stay in Canberra, Mike had access to Adrian Horridge’s fabulous “meccano set” of small optical components from the German company Spindler and Hoyer (which came in a gorgeous felt-lined polished wooden case). Using these, Mike built a very clever benchtop ophthalmoscope to peer into the superposition eyes of moths and butterflies. This device allowed Mike to use the eye’s own optics to image the retina. When he looked at the retinas of day-flying skipper butterflies and agaristid moths (which paradoxically have superposition eyes despite their diurnal activities), he discovered that he could clearly see their rhabdoms,^2^ implying that the optics of their eyes were producing very sharp images. In fact, calculations revealed that such image quality is only possible if their eyes are free from optical aberrations and limited only by diffraction (which all eyes, no matter how sharp, cannot avoid). Thus, the eyes of skippers and agaristids produce the crispest retinal images that the optics of any eye can provide! Mike’s discovery forever dispelled the idea that superposition eyes were necessarily poorly focussed. However, these eyes, despite being of the superposition type, are adapted for bright light and as such are much smaller, and have much smaller superposition apertures, than those of their nocturnal relatives. My own later work on dung beetles showed that in the hunt for photons at night, nocturnal species with larger eyes and wider apertures are unable to escape aberrations, and thus sacrifice spatial resolution for sensitivity, a classic trade-off in eye design that has fascinated me throughout my life.

One of my other *JCPA* heroes from that time was Dan-Eric Nilsson. Little did I know, while I was reading his amazing optical accounts of crustacean compound eyes in *JCPA* (e.g. Nilsson and Nilsson 1981; Nilsson and Odselius 1981; Nilsson 1990), that Dan would soon become one of my closest friends and profoundly change the course of my life. In 1988, toward the end of my PhD, I made my first trip to Europe and my first port of call was Lund—to visit Dan. The rest is history. After Dan successfully applied for postdoc funding, I arrived in Lund in August 1990 and never left! He and I started and grew the Lund Vision Group, which following Dan’s retirement in 2022, I now lead.

But what really grabbed my attention back in Canberra—before I even knew him—was Dan’s remarkable discovery of afocal optics in butterfly apposition eyes, the likely evolutionary link between apposition and superposition eyes (Nilsson et al. 1988). In nearly all apposition eyes the crystalline cone in each ommatidium is little more than a watery optical spacer between the image-forming corneal facet lens and the tip of the rhabdom (where the light is focussed). But in certain butterflies, the crystalline cone is still mostly watery, but just distal to the point where it adjoins the tip of the rhabdom it narrows into a much denser stalk-like tip (called the cone stalk) within which a powerful radial gradient of refractive index is present. The facet lens brings light rays to an intermediate focus within the distal region of the cone stalk and these rays are then recollimated by the refractive-index gradient, which acts like a powerful second lens. Compared to a regular (“focal”) apposition eye, and without going into the details of the waveguide optics involved, the cone-stalk turns out to simultaneously sharpen spatial resolution and boost light capture—an impressive adaptation that apparently defies the classical trade-off between resolution and sensitivity!

*JCPA* also published many of the most important discoveries in photoreceptor optics, all of which opened my eyes when I was a student. These include the first measurement of the refractive index of an insect rhabdom (Stavenga 1974), the earliest theoretical treatments of the function of the fused rhabdom (Snyder and Pask 1972a; Snyder et al. 1973), and the ability of the rhabdom to code spatial (Snyder and Pask 1972b), spectral (Snyder and Pask 1972c, 1973) and polarisation information (Snyder 1973; Snyder and Sammut 1973; Menzel and Snyder 1974; Snyder and Laughlin 1975). In parallel to these paradigm-shifting studies, Doekele Stavenga published a series of highly influential papers on the optical effects of the moveable screening pigments (Stavenga and Kuiper 1977; Stavenga et al. 1977, 1979; Roebroek and Stavenga 1990). And in a beautiful series of studies, Doekele’s students studied the waveguiding properties of the extremely thin rhabdomeres of flies (which have a diameter only twice the wavelength of green light), showing how their spatial and spectral properties are entirely determined by the numbers and types of waveguide modes they propagate, and by the movements of screening pigments (van Hateren 1984; Smakman et al. 1984).

Arguably though, the paper that has had the greatest long-term influence on my intellectual development was Rüdiger Wehner’s fabulous *JCPA* paper on sensory matched filters (Wehner 1987). Even before I saw this paper, I was already awe-struck by Rüdiger’s work on the navigational wonders of the desert ant *Cataglyphis* (his must-read book *Desert Navigator* (Wehner 2020) exquisitely summarises his life’s work on this ant over 5 decades). I first met Rüdiger in Zürich in 1988, on that same European tour when I first met Dan-Eric Nilsson. It was late November, and Zürich was covered in a thick coat of fresh snow. It was stunningly beautiful. When we walked with our backpacks to what I assumed would be a hostel, we instead came to a gorgeous antique-filled hotel. I could not believe my eyes. Rüdiger was treating me—a lowly PhD student—to the best hotel I had ever stayed in (and for me it still remains one of the finest). This level of hospitality and care only continued throughout my visit. Despite he being a towering intellectual giant with a direct academic line to Karl von Frisch, and me a nervous PhD student from the antipodes, he and I struck up a genuine rapport, and we have remained firm friends ever since.

What Rüdiger Wehner elegantly showed us in his 1987 paper is that in a world of effectively infinite sensory information, the senses of animals must act as filters, accepting essential sensory signals and rejecting other non-essential signals. But more than this, he explains that most sensory filters are actually pre-adapted—or matched—to the characteristics of the information they are tuned to detect. By matching the properties of neurons, circuits and sensory structures to the characteristics of the most crucial sensory stimuli that need to be detected, these stimuli can be directly and reliably extracted for further processing. All other sensory stimuli—having little consequence for the animal’s chances of reproduction and survival—are simply suppressed or filtered out altogether. To see “the world through such a matched filter”, to quote Rüdiger himself, “severely limits the amount of information the brain can pick up from the outside world, but it frees the brain from the need to perform more intricate computations to extract the information finally needed for fulfilling a particular task”. In example after example, both vertebrate and invertebrate and using a variety of senses, he shows how matched filtering is used by animals to solve complex tasks in a highly efficient manner. Matched filters also save energy, because by “severely limiting information picked up by the brain”, the energetic costs that would have been associated with coding superfluous information are effectively eliminated. With this paper, I suddenly understood that the ecologies of animals do not simply shape their senses, but actually hone them to detect only the specific signals crucial for reproduction and survival, saving nervous tissue and thus precious energy. These ideas completely revolutionised the way I understood sensory evolution. Matched filters crept into my teaching, inspired my research and finally drove the creation of an entire book (von der Emde and Warrant 2016).

In my mind, I often travel back to those halcyon days at Gosford High School and ponder my fateful decision to scratch physics from my education at the age of 15. What would have happened if I had not been so interested in art? I would have certainly studied physics just like all my friends. And like them, I probably would have hated it (the teacher was dreadful). No doubt I would have gone on to study entomology as planned. But what then? A career in agriculture fighting pests? Maybe a PhD in some applied aspect of entomology? It actually makes me wince to think about it. Without that passion for art, the great riches of physics that were revealed to me for precisely *not* studying physics at school, and the thrills of studying “entomological physics” ever since, would have been blocked forever. Who could have imagined how such a simple (and apparently rash) childhood decision could have sealed my entire fate? Nor how the care, interest and encouragement of professors like David Sandeman at the University of New South Wales would steer me in the right direction? His advice to go to Canberra could not have been better. If there was any question remaining in my mind that entomology and physics were a crazy mix, it was thoroughly dispelled in Canberra. And it became overwhelmingly clear to me that the past editors of the *Journal of Comparative Physiology* did not think the mix was so crazy either—the journal was (and still is) packed with “entomological physics”! I have since met nearly all of my early “*JCPA* heroes”, and all of them are now friends, some very close friends. It is amazing to think that a seemingly foolhardy decision as a 15-year-old, and my discovery of the biological wonders held in this remarkably formative journal, not only decided and steered my research trajectory but also formed and enriched my entire life.

Viva *Journal of Comparative Physiology A* ! After your long and profound influence on my life I am now proud to serve on your Editorial Board and finally give something back. May your next 100 years be just as formative for the next generation of neuroethologists as your past 40 years have been for me!

## Footnotes

1 The tapering cylinder-like optical building blocks of a compound eye, consisting of set of lenses (the corneal facet lens and the underlying crystalline cone) that focus light onto (typically) 8 or 9 photoreceptor cells. Moveable screen pigments may ensheath the entire ommatidium so that it is optically isolated from its neighbours (as in apposition compound eyes). Each ommatidium receives light from a narrow region (cf. pixel) of visual space, with neighbouring ommatidia sampling neighbouring regions of space.

2 The rhabdom is formed from the light-sensitive regions of each photoreceptor cell within the ommatidium. In superposition eyes they adjoin to create a “fused rhabdom”, a rod-like structure that absorbs and transduces the incoming light.
